# Correlation of placental microbiota with fetal macrosomia and clinical characteristics in mothers and newborns

**DOI:** 10.18632/oncotarget.19319

**Published:** 2017-07-18

**Authors:** Jia Zheng, Xin-Hua Xiao, Qian Zhang, Li-Li Mao, Miao Yu, Jian-Ping Xu, Tong Wang

**Affiliations:** ^1^ Department of Endocrinology, Key Laboratory of Endocrinology, Ministry of Health, Peking Union Medical College Hospital, Diabetes Research Center of Chinese Academy of Medical Sciences & Peking Union Medical College, Beijing 100730, China

**Keywords:** fetal macrosomia, placenta, microbiota, 16S rRNA gene, clinical characteristics

## Abstract

Substantial studies indicated that fetal macrosomia was associated with detrimental pregnancy outcomes, and increased susceptibility to metabolic diseases in later life. However, investigations into the association between placental microbiota and fetal macrosomia are limited. We aimed to profile the placental microbiota of fetal macrosomia and study whether they relate to clinical characteristics. Placenta samples were collected from fetal macrosomias and newborns with normal birth weight. The clinical characteristics, umbilical cord blood parameters were measured, and placental microbiota were sequenced and further analysed. The clinical characteristics of infants and mothers and umbilical cord blood parameters were significantly different between macrosomias and controls. The relative abundance of microbiota sequences revealed that microbial structures of the placenta differed significantly between macrosomia and controls. Regression analysis showed a cluster of key operational taxonomic unit (OTUs), phyla and genera were significantly correlated with body length, ponderal index and placenta weight, body weight increase during pregnancy of mothers, and cord blood IGF-1 and leptin concentrations. In conclusion, our study for the first time explored the relationship between placental microbiota profile and fetal macrosomia. It is novel in showing that a distinct placental microbiota profile is present in fetal macrosomia, and is associated with clinical characteristics of mothers and newborns.

## INTRODUCTION

Fetal macrosomia has a birth weight of more than 4000 grams, which can increase the risks of poor pregnancy outcome in mothers and newborns [1]. The prevalence of macrosomia ranged from 5% to 20%, and has increased by 15–25% during the past three decades [2]. Possible maternal complications of fetal macrosomia might include: operative vaginal delivery, C-section needed, chorioamnionitis, prolonged hospital stay, postpartum infection and haemorrhage [3] Fetal macrosomia is associated with an increased risk of severe perineal lacerations by 4.5 times [4]. Fetal macrosomia could put the baby at increased risk of injury during birth, such as shoulder dystocia, clavicle fracture, birth asphyxia, and perinatal mortality [5, 6]. Infants weighing over 4500 g carries a 21-fold elevated risk of shoulder dystocia [7]. For the long term effects, infants born with macrosomia may be more likely to develop obesity, type 2 diabetes and cardiovascular diseases in adulthood, known as “fetal programming hypothesis” [8]. Koyanagi et al. showed that an increasing rate of fetal macrosomia was in parallel with rises in obesity and diabetes for women in the child-bearing age [9]. Thus, macrosomia has been identified as a worldwide problem [10].

In 2012, the Human Microbiome Project Consortium for the first time showed that healthy human microbiome exists across multiple body sites, such as mouth, stomach, gut, urogenital tract, lungs, stool and skin [11]. Qin et al. showed that gut microbial markers were closely related with T2DM [12]. However, information about the placental microbiota structure is poorly understood, especially that placenta is critical to fetal growth, development and survival during pregnancy [13]. In recent years, it has become clear that the placenta is not a “sterile” organ [14], but rather has its own endogenous microbiome. The composition of the placental microbiome is distinct from that of the vagina and has been reported to be composed of nonpathogenic commensal microbiota and most akin to the human oral microbiome [15, 16], and it was also associated with human health and pregnancy outcomes. Preterm placental membranes showed distinct microbial species, compared with term [17]. Kathleen et al. demonstrated that the microbial structure in placentas from preterm births was associated with excess gestational weight gain [18]. Our previous data showed that there was significant difference in placenta microbiota composition between full-term low birth weight neonates and controls [19]. However, the explicit roles of placental microbiota on fetal macrosomia are still unclear. Therefore, our objective was: 1) to investigate the structure and diversity of placental microbiota in fetal macrosomia; 2) to determine whether they relate to clinical characteristics of mothers and infants, and umbilical cord blood biochemical parameters.

## RESULTS

### Clinical characteristics and umbilical cord blood parameters

The clinical characteristics of infants with umbilical cord blood parameters, and mothers were shown in Table 1 and Table 2, respectively. For the infants, it indicated that birth weight (4441.50±199.17 vs. 3147.00±78.32, *P* < 0.001), body length (52.20±1.32 vs. 49.20±1.03, *P* < 0.001), ponderal index (31.02±2.61 vs. 26.47±1.57, *P* < 0.001) and placenta weight (941.00±114.26 vs. 593.75±69.01, *P* < 0.001) were statistically increased in fetal macrosomia, compared with newborns with normal birth weight. There was no significant difference in head circumference and gestational age (*P* > 0.05) between macrosomia and control groups (Table 1). Moreover, we measured insulin, leptin and IGF-1 levels in cord blood. It showed that leptin (14.84±6.71 vs. 7.50±3.82, *P* = 0.008) and IGF-1 (93.20±56.88 vs. 51.99±22.19, *P* = 0.047) concentrations were elevated in fetal macrosomia. There was a tendency to have higher insulin concentrations in fetal macrosomia, compared to newborns with normal birth weight (*P* = 0.086) (Table 1).

**Table 1:** Comparison of clinical characteristics of infants between macrosomia and control groups

Groups	Birth Weight (g)	Body Length (cm)	Ponderal Index (kg/m^3^)	Head Circumference (cm)	Placenta Weight (g)	Gestational Age (Weeks)	Insulin (μU/mL)	Leptin (ng/mL)	IGF-1 (ng/mL)
**Control**	3147.00±78.32	49.20±1.03	26.47±1.57	33.87±0.98	593.75±69.01	39.03±0.75	13.14±5.21	7.50±3.82	51.99±22.19
**Macrosomia**	4441.50±199.17	52.20±1.32	31.02±2.61	34.75±1.39	941.00±114.26	39.29±1.22	20.67±12.03	14.84±6.71	93.20±56.88
**P value**	< 0.001***	< 0.001***	< 0.001***	0.118	< 0.001***	0.576	0.086	0.008**	0.047*

Data reported as the mean ± standard deviation (S.D.). n=10, in each group. *P < 0.05, **P < 0.01, ***P<0.001 macrosomia vs. control group. IGF-1, insulin-like growth factor-1.

**Table 2:** Clinical characteristics of mothers

Groups	Age	BW (1st trimester)	BMI (1st trimester)	BW (3rd trimester)	BMI (3rd trimester)	BW increase	BMI increase
**Control**	32.20±5.07	53.50±7.38	19.81±1.72	69.50±7.40	25.77±1.50	16.00±3.57	5.96±1.33
**Macrosomia**	30.60±3.13	60.70±7.84	22.76±2.93	81.55±7.13	30.62±2.90	20.85±4.72	7.86±1.98
**P value**	0.407	0.049*	0.013*	0.002**	0.000***	0.018*	0.021*

Data reported as the mean ± standard deviation (S.D.). n=10, in each group. *P < 0.05, **P < 0.01, ***P < 0.001 macrosomia vs. control group. BW, body weight; BMI, body mass index.

For the mothers, there is no difference of maternal age between the two groups. We found both body weight and BMI at the beginning of 1^st^ trimester (60.70±7.84 vs. 53.50±7.38, *P* = 0.049 and 22.76±2.93 vs. 19.81±1.72, *P* = 0.013, respectively) and the end of 3^rd^ trimester (81.55±7.13 vs. 69.50±7.40, *P* = 0.002 and 30.62±2.90 vs. 25.77±1.50, *P* = 0.000, respectively) were higher in macrosomia group. Both body weight (20.85±4.72 vs. 16.00±3.57, *P* = 0.018) and BMI (7.86±1.98 vs. 5.96±1.33, *P* = 0.021) increase during pregnancy were higher in macrosomia, compared to control group (Table 2).

### Validation of the sequencing accuracy and community diversity

To profile the differences of birth weight on placenta microbiota structure, we performed Illumina MiSeq sequencing of bacterial 16S rRNA gene V3 region for 20 placenta samples collected from the two groups. The estimated sample coverage (Good’s coverage) was about 99% and the correlation between duplicate samples was more than 99.5%, which indicated the accuracy and reproducibility of sequencing was reliable. A total of 720817 sequences were obtained and each sample provided 36040±4067 sequences on average. The high quality sequence count (36303±4137 vs. 32475±3609) and operational taxonomic unit (OTU) count (153.8±59.1 vs. 176.5±28.8) were similar in the two gropus. No significant difference of estimated OTU richness (Chao) was observed between the two groups. However, significant differences were observed in the estimators of community diversity indices including Shannon (2.76±0.52 vs. 1.97±0.66, *P* = 0.02) and simpson (0.12±0.07 vs. 0.34±0.19, *P* = 0.007) between the two groups, indicating higher micorbiota diversity in placenta from macrosomia (Table 3).

**Table 3:** Sequencing data summary and community diversity

	Control	Macrosomia	P value
**Sequences**	32475±3609	36303±4137	0.74
**OTUs**	176.5±28.8	153.8±59.1	0.34
**Chao**	51.06±18.68	58.26±23.06	0.49
**Shannon**	1.97±0.66	2.76±0.52	0.02*
**simpson**	0.34±0.19	0.12±0.07	0.007**

Data represents as mean ± standard deviation (S.D.) Statistical analyses were performed with Mann-Whitney U test between the two groups. The number of OTUs, richness estimator Chao, and diversity estimator Shannon were calculated at 3% distance. n=10, in each group. *P < 0.05, **P < 0.001 macrosomia vs. control group. OTU, operational taxonomic unit.

### Overall structural changes of placental microbiota in response to birth weight

**Figure 1:**
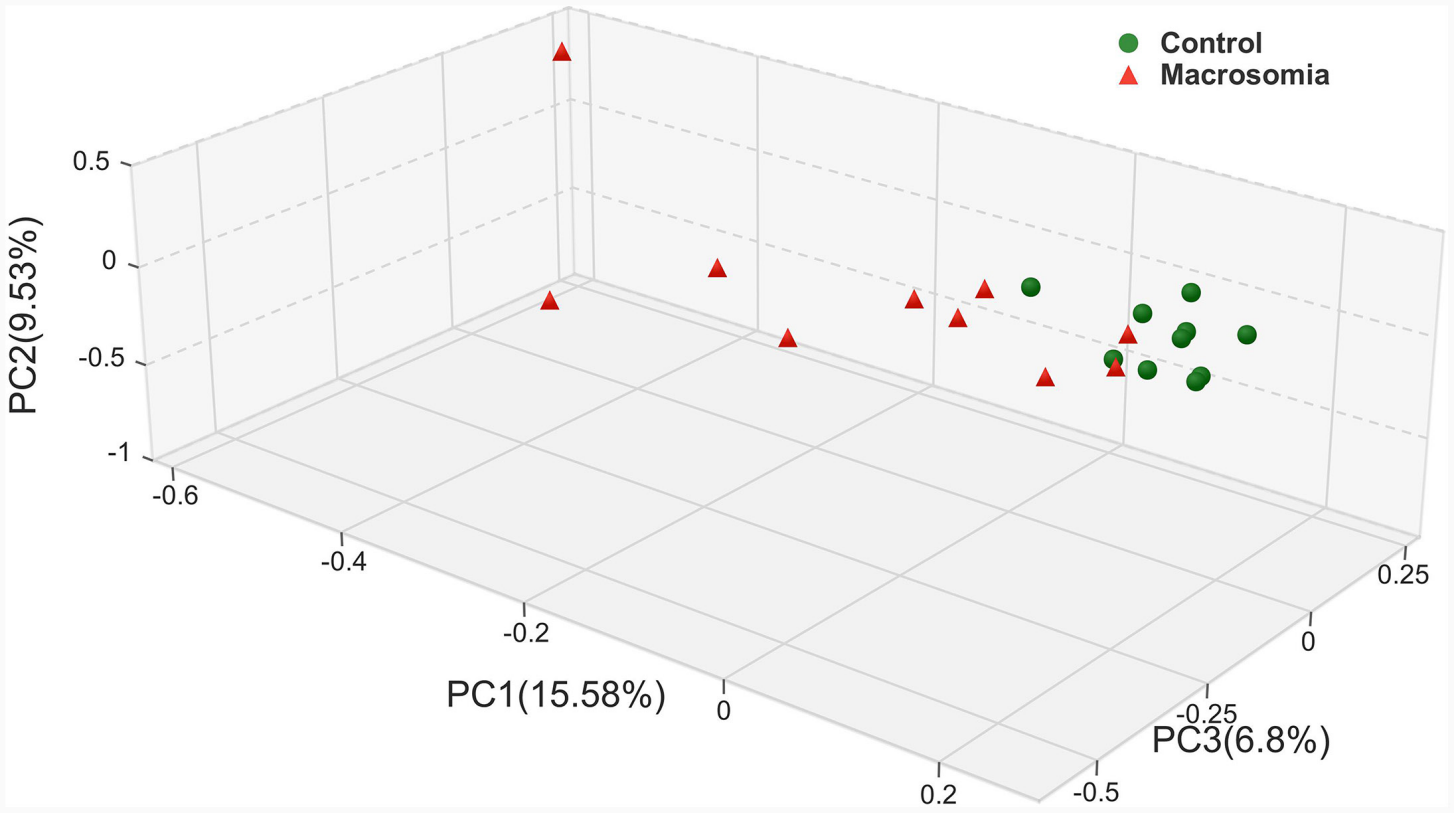
Principal coordinate analysis plots in placenta microbiota of newborns Weighted UniFrac PCoA plot based on OTU abundance. Each point represents the placenta microbiota of a newborn, with macrosomia group (red triangle) and control group (green circle). n=10 in each group.

Closer analyses of placental microbiota differences by virtue of birth weight were performed. Principal coordinates analysis (PCoA) of sequencing data showed significantly separate clustering of the placental microbiota structure between fetal macrosomia and control groups, with main principal component (PC) scores: PC1 = 15.58%, PC2 = 9.53% and PC3 = 6.8%, demonstrating a different clustering according to birth weight (Figure 1).

**Figure 2:**
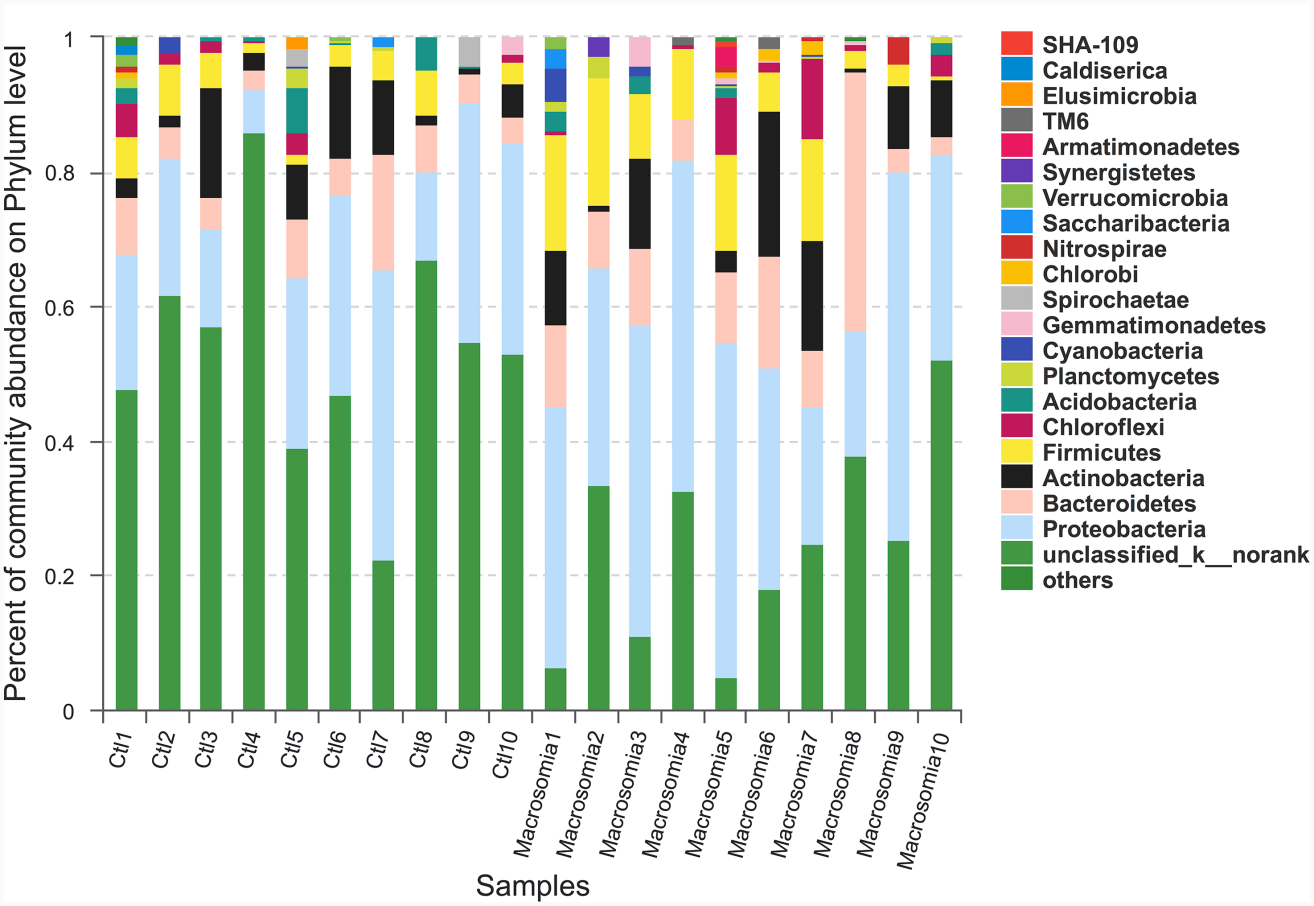
Community bar-plot analysis shows relative abundance of placenta microbiota in each sample at the phylum level n = 10, in each group. Ctl: control.

**Figure 3:**
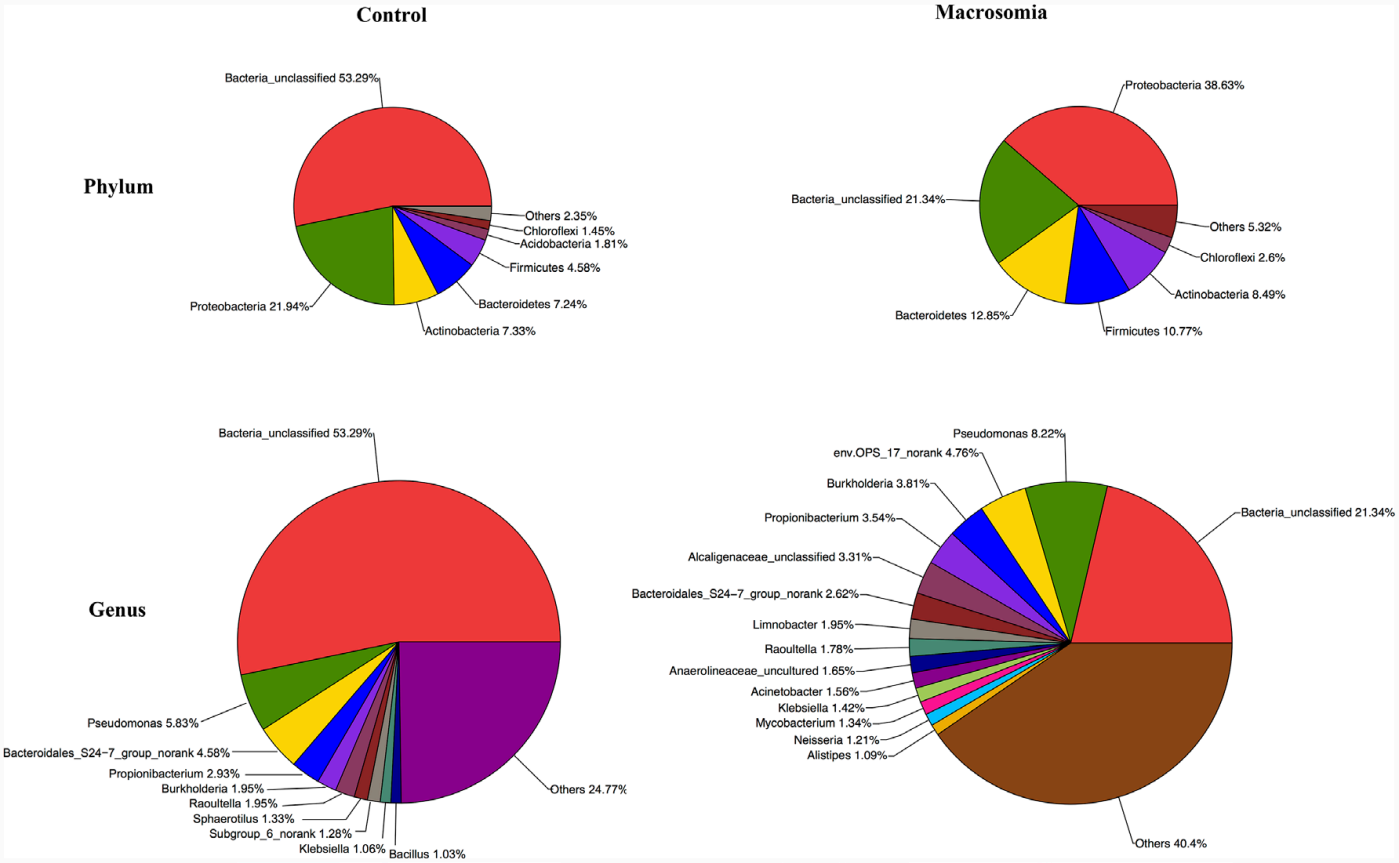
Community analysis pie-plot shows the overall microbiota structure for each group at the phylum and genus levels n = 10, in each group.

**Figure 4:**
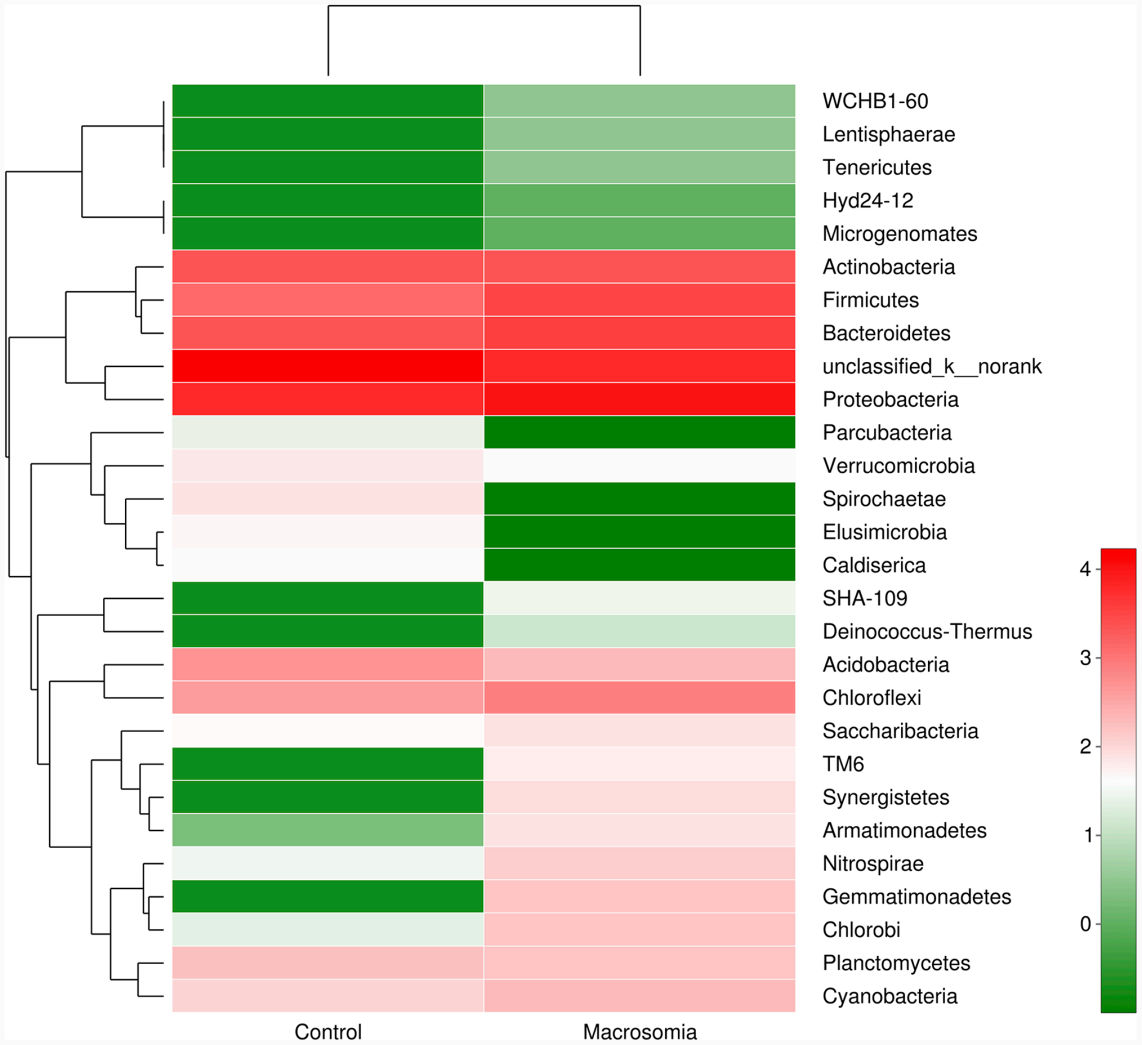
Heatmap analyses of abundant phylum in each group The y axis is a neighbor-joining phylogenetic tree, each row is a different phylotype. The color of the spots in the right panel represents the mean relative abundance of the phyla in each group. n = 10, in each group.

Community bar-plot analysis displays relative levels of placenta microbial community in all the 20 placenta samples at the phylum level (Figure 2). Community analysis pie-plot exhibits the overall microbial community structures in fetal macrosomia and control groups (Figure 3). The dominant phyla of the two groups were: Proteobacteria, Bacteroidetes, Firmicutes, and Actinobacteria. The proportion of Proteobacteria, Bacteroidetes, Firmicutes, and Actinobacteria were increased in macrosomia group, compared to controls. The dominant genus of the placental microbiota was Pseudomonas. The relative percentage of Pseudomonas composition was higher in macrosomia group. The heatmap profile also indicated that microbial community structures in placentas had a marked difference between fetal macrosomia and normal birth weight (Figure 4).

### Placental microbiota composition differ between macrosomias and controls

**Figure 5:**
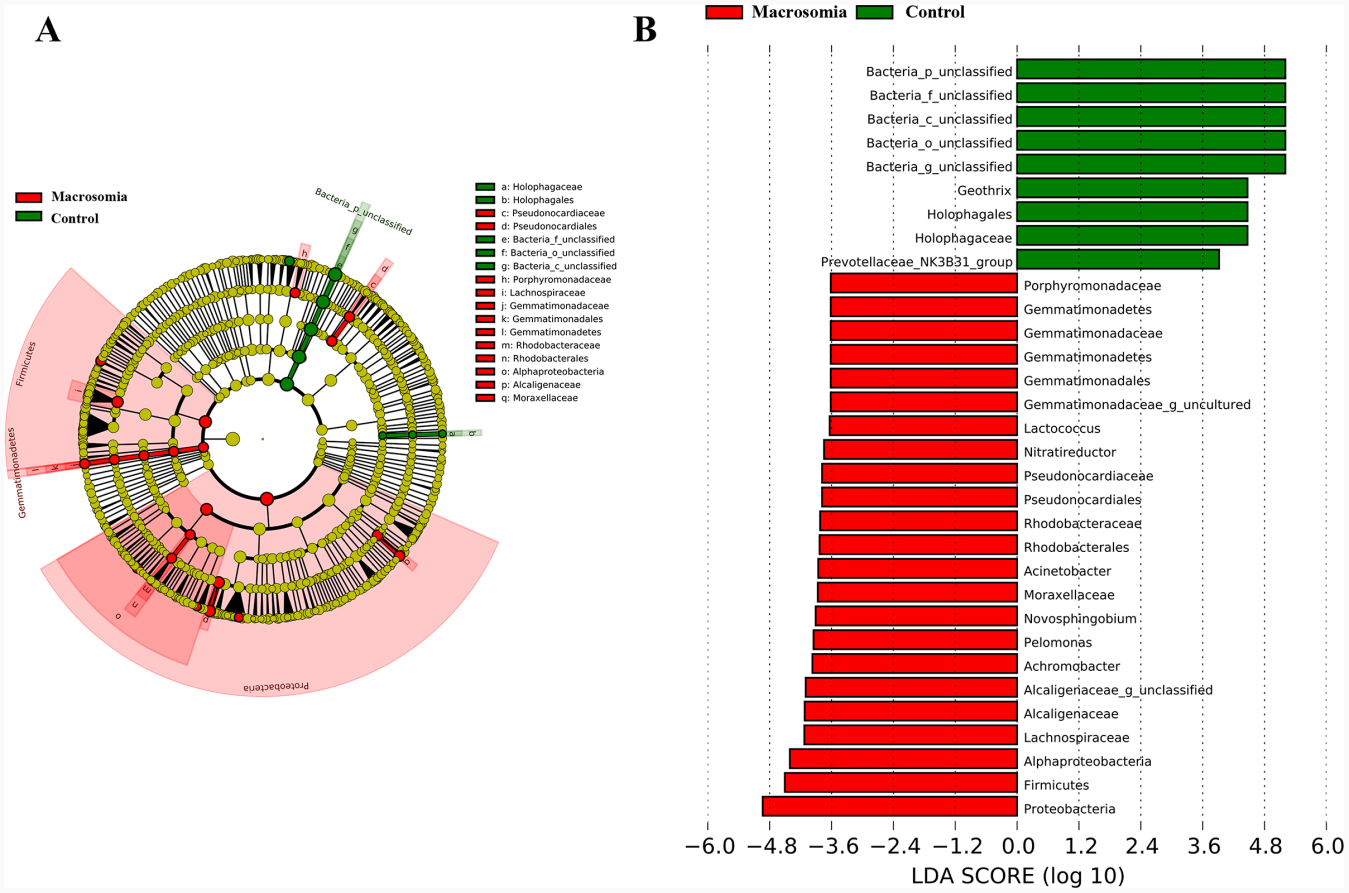
Bacterial difference identification in placental microbiota in macrosomia vs. control groups **(A)** Taxonomic representation of statistically and biologically consistent differences between macrosomia and control groups. Differences are represented by the color of the most abundant class (red: macrosomia group; yellow: nonsignificant; and green: control group). The diameter of each circle is proportional to the taxon’s abundance. **(B)** Linear discriminant analysis score, used to build the cladogram in macrosomia (red) vs. control groups (green). Histogram of the LDA scores for differentially abundant genera. LDA scores were calculated by LDA effect size, using the linear discriminant analysis to assess effect size of each differentially bacterial taxa (red: macrosomia group; green: control group). The cladogram is displayed according to effect size. n=10, in each group. LDA: linear discriminant analysis.

**Figure 6:**
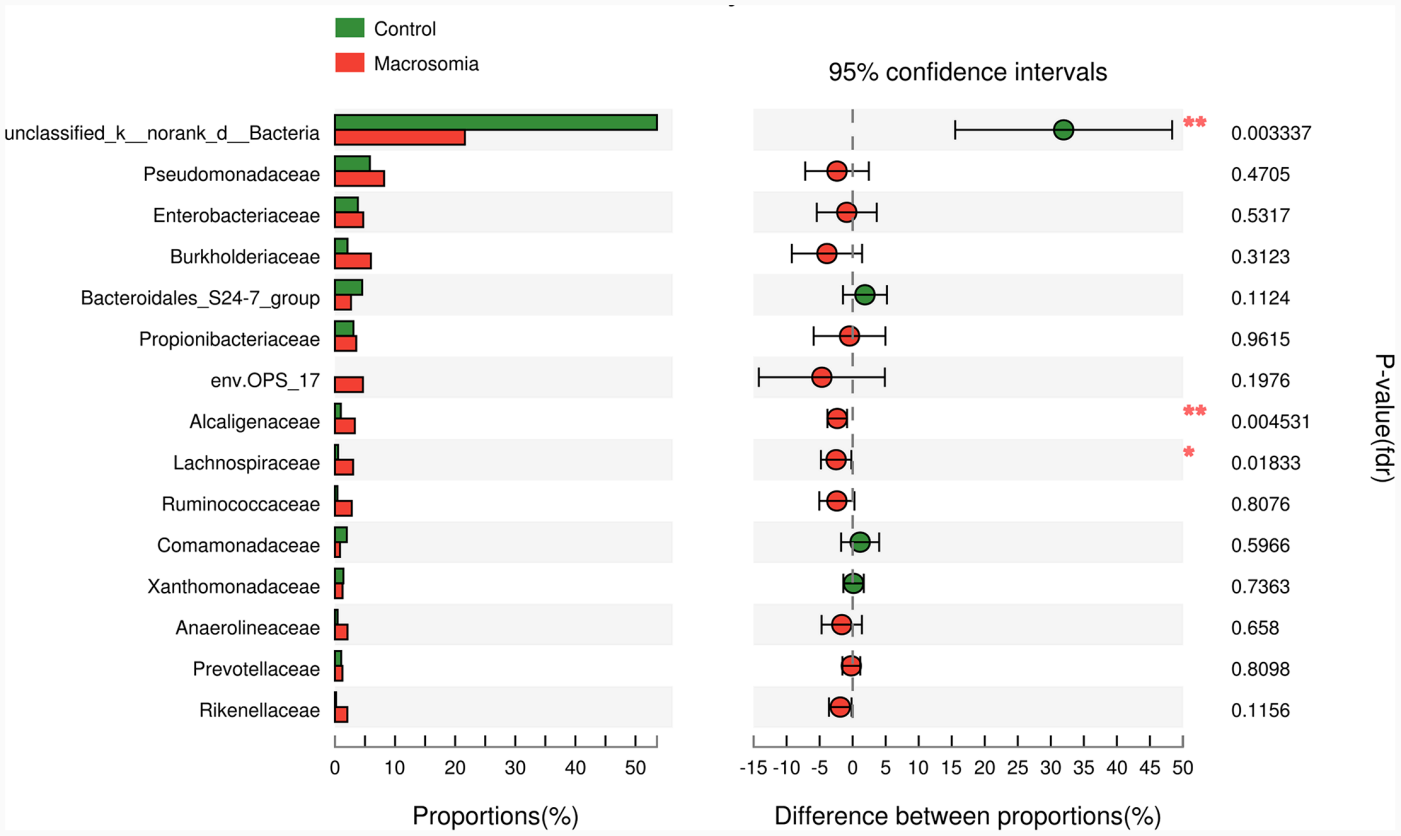
Phylotypes significantly different between macrosomia and control groups at family level Data of macrosomia and control groups were showed as relative abundance (%) of phylum and family in each group. Statistical analysis was performed by the Mann-Whitney U test. n=10, in each group. *P <0.05, **P <0.001 macrosomia vs. control group.

We further investigated whether or not certain placental microbiota structure correlated with fetal macrosomia. A cladogram displayed the all the different microbiota structure from phylum and species level (Figure 5A). The greatest differences in placental microbiota taxon between macrosomia and controls, according to the Linear Discriminant Analysis (LDA) score, are displayed in Figure 5B. The specific phylum, family and genus differences are shown in Table 4, Figure 6 and Supplementary Table 2, respectively.

**Table 4:** Phylotypes significantly different at the phylum level

Taxonomy	Control (proportions)	Macrosomia (proportions)	P value
**Bacteria_unclassified**	53.29%	21.34%	0.003**
**Proteobacteria**	21.94%	38.63%	0.024*
**Firmicutes**	4.58%	10.77%	0.039*
**Gemmatimonadetes**	0.00%	0.61%	0.045*
**Bacteroidetes**	7.24%	12.85%	0.112
**Nitrospirae**	0.11%	0.54%	0.196
**Deinococcus-Thermus**	0.00%	0.04%	0.198
**Synergistetes**	0.00%	0.28%	0.198
**TM6**	0.00%	0.24%	0.198
**Caldiserica**	0.17%	0.00%	0.346
**Elusimicrobia**	0.18%	0.00%	0.346
**Spirochaetae**	0.30%	0.00%	0.346
**Acidobacteria**	1.81%	0.78%	0.359
**Hyd24-12**	0.00%	0.01%	0.409
**Lentisphaerae**	0.00%	0.01%	0.409
**Microgenomates**	0.00%	0.00%	0.409
**SHA-109**	0.00%	0.12%	0.409
**Tenericutes**	0.00%	0.02%	0.409
**Parcubacteria**	0.11%	0.01%	0.477
**Cyanobacteria**	0.30%	0.77%	0.495
**Saccharibacteria**	0.15%	0.31%	0.664
**Armatimonadetes**	0.01%	0.28%	0.746
**Chlorobi**	0.09%	0.54%	0.839
**Verrucomicrobia**	0.27%	0.17%	0.858
**Actinobacteria**	7.33%	8.49%	1.000
**Chloroflexi**	1.45%	2.60%	1.000
**Planctomycetes**	0.66%	0.57%	1.000
**WCHB1-60**	0.00%	0.02%	1.000

Data of macrosomia and control groups were showed as relative abundance (%) of phylum and family in each group. Statistical analysis was performed by the Mann-Whitney U test adjusted for multiple testing. n=10, in each group. *P < 0.05, **P < 0.001 macrosomia vs. control group.

We further compared the relative levels of placental microbiota between macrosomia and control groups at phylum-, family- and genus-levels. Proteobacteria, Firmicutes and Gemmatimonadetes phyla were statistically increased (*P < 0.05), while Bacteria_unclassified (**P < 0.01) was statistically decreased in macrosomia, compared to controls (Table 4). At the family level, the percentages of Alcaligenaceae and Lachnospiraceae were higher (**P < 0.01 and *P < 0.05, respectively) and Bacteria_unclassified was lower in macrosomia, compared to controls (**P < 0.01) (Figure 6). For the comparisons of genus composition, 12 genera were differed between the two groups, with the percentages of Novosphingobium, Achromobacter, Acinetobacter, Paracocccus, Pseudonocardia, Woodsholea, Nitratireductor, Pelomonas and Alcaligenaceae_unclassified increased in macrosomia. Conversely, the relative levels of Geothrix, Prevotellaceae_NK3B31_group and Bacteria_unclassified were statistically decreased in macrosomia, compared to controls (Supplementary Table 2). Therefore, these data demonstrated that placental microbial community composition differed significantly between macrosomias and controls.

### Correlation between overall placenta microbiota structure and clinical parameters

**Figure 7:**
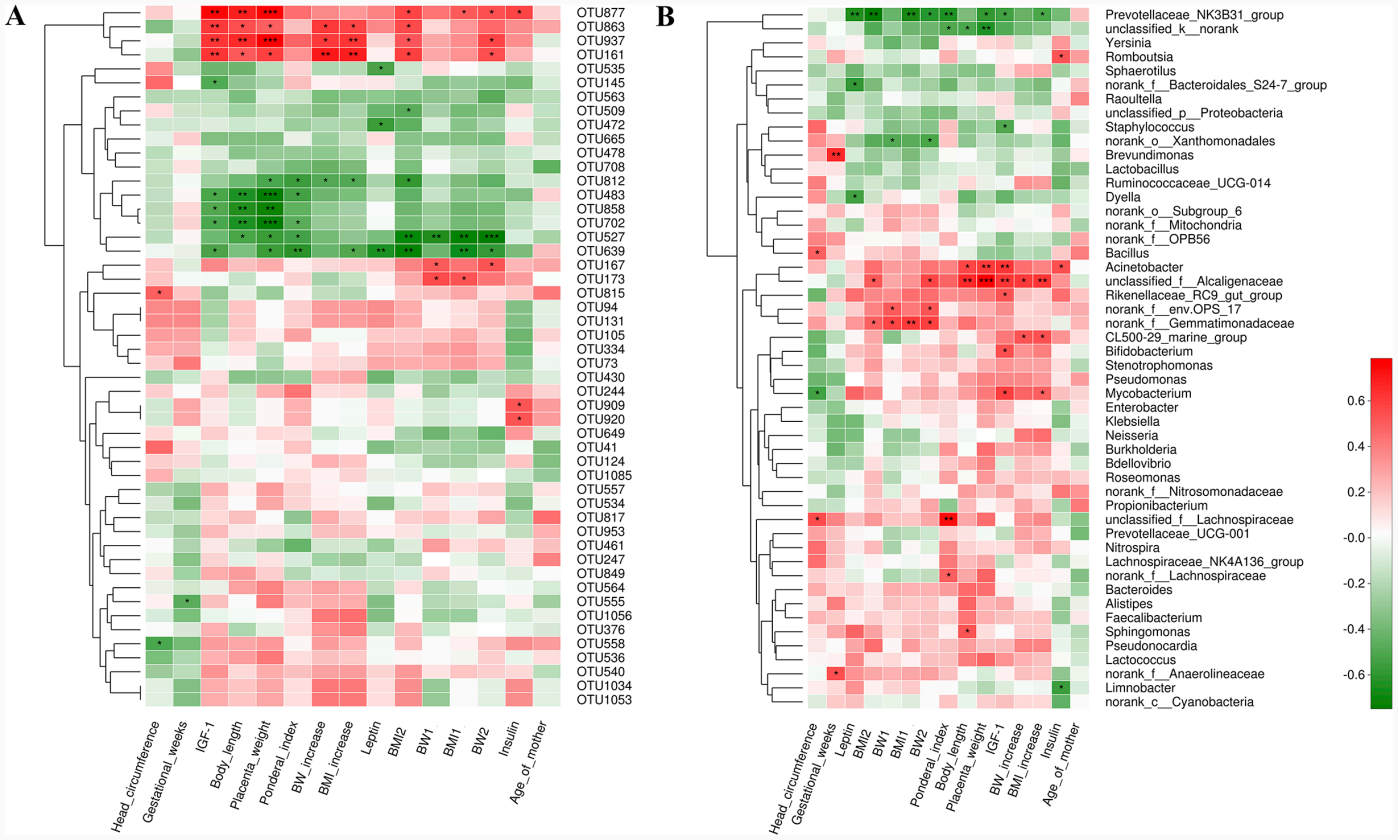
Correlation analyses between relative abundance (%) of placental microbiota and clinical parameters at the OTU and genus level **(A)** OTUs that were significantly correlated with clinical parameters. **(B)** Correlation analysis between identified genera and clinical parameters. Correlation analyses between relative abundance (%) of specific OTU and genus and clinical parameters were performed by using Spearman’s correlation analyses. n=10, in each group. (*) represents the specific OTU and genus whose abundance were significantly correlated with certain clinical parameters. The color of the spots in the right panel represents R-value of Spearman’s correlation between the OTU and genus and clinical parameters. OTU, operational taxonomic unit; BW, body weight; BMI, body mass index; BMI1, BMI at the beginning of 1st trimester; BMI2, body mass index at the end of 3rd trimester; BW1, body weight at the beginning of 1st trimester; BW2, body weight at the end of 3rd trimester; IGF-1, insulin-like growth factor-1.

Our next goal was to identify specific placental bacteria that potentially are associated with certain clinical characteristics and umbilical cord blood parameters. Spearman’s correlation analysis was performed between the top 50 OTUs according to the relative abundance that were changed by birth weight and specific clinical parameters in all the mothers and infants. In total, 21 OTUs (herein designated ‘key’ OTUs) were significantly correlated with at least one clinical parameter. Three to four OTUs were positively correlated with body length and placenta weight, body weight and BMI at the end of 3^rd^ trimester, body weight and BMI increase during pregnancy of mothers, and umbilical cord blood IGF-1 concentrations. Three OTUs were positively correlated with umbilical cord blood insulin concentrations. Four to six of these key OTUs were correlated with decreased body length, placenta weight and ponderal index in infants, as well as umbilical cord blood IGF-1 concentrations. One to two key OTUs were negatively correlated with body weight and BMI at the beginning of 1^st^ and the end of 3^rd^ trimester, body weight and BMI increase during pregnancy in mothers. Three OTUs were negatively correlated with umbilical cord blood leptin concentrations. No OTUs were correlated with gestational age and age of the mothers (Figure 7A).

At the phylum level, higher relative abundance of Proteobacteria in placental microbiota was associated with ponderal index and placenta weight. In addition, Bacteroidetes was positively correlated with body weight at the beginning of 1st and the end of 3rd trimester. Higher relative abundance of Gemmatimonadetes was associated with higher body weight and BMI at the beginning of 1st and the end of 3rd trimester (Supplementary Figure 1). At the genus level, 22 genera were significantly correlated with at least one clinical parameter. Of note, Prevotellaceae_NK3B31_group was negatively correlated with several parameters, including ponderal index, placenta weight, body weight and BMI at the end of 3^rd^ trimester and BMI increased. Acinetobacter was positively correlated with body length and placenta weight. Next, we focused on umbilical cord blood biochemical parameters. It showed that Acinetobacter, Bifidobacterium and Mycobacterium genera were correlated with elevated IGF-1 level, while prevotellaceae_NK3B31_group and Staphylococcus were correlated with decreased IGF-1 level. Prevotellaceae_NK3B31_group, Dyella and norank_f__Bacteroidales_S24-7_group were correlated with decreased leptin level. Romboutsia and Acinetobacter were positively, and Limnobacter was negatively associated with insulin level (Figure 7B). Thus, the correlations between specific OTUs/phyla/genera and cord blood insulin, IGF-1 and leptin level may help to explain their roles in the development of fetal macrosomia.

## DISCUSSION

Substantial studies indicated that macrosomia has multiple detrimental impacts, including the short-term outcomes and long-term effects. Our previous study showed that birth weight determines the risks of metabolic syndrome in adult life for the time in a Chinese population [20, 21]. Moreover, it showed that increasing prevalence of macrosomic births was paralleled in rise in diabetes and obesity in women [9]. Recently, increasing studies demonstrated the existence of a placental microbiota and it was associated with pregnancy outcomes and human health [15–18]. More recently, the maternal-fetal transmission of microorganisms and microbial colonization of the fetal organism before birth has been suggested. Therefore, our objective was to investigate the difference of placental microbial community between fetal macrosomia and newborns with normal birth weight. Consistent with previous studies [15, 18], nonpathogenic commensal species were mainly detected in the placental microbiota, and the most abundant was Proteobacteria. However, our study for the first time showed that placenta with macrosomia have distinct microbial community compositions, with higher relative levels of Proteobacteria and Firmicutes genera.

Similar to a couple of other studies, it showed that pre-pregnancy BMI, gestational week, and gestational weight gain were strongly associated with fetal macrosomia [22, 23]. In our study, we found both body weight and BMI increases during pregnancy were higher in macrosomia. We further investigated whether placental microbiota was associated with anthropometric measurements, and cord blood biochemical parameters, including insulin, leptin and IGF-1. We found that in macrosomia, higher relative abundance of Proteobacteria phylum in placental microbiota was correlated with placenta weight and ponderal index. Acinetobacter genus was positively correlated with placenta weight, ponderal index, umbilical cord blood IGF-1 and inslin levels. In addition, Bacteroidetes phylum and norank_f_env.OPS_17 genus was positively correlated with body weight at the beginning of 1st and the end of 3rd trimester. Higher relative level of Gemmatimonadetes phylum was correlated with higher body weight and BMI at the beginning of 1st and the end of 3rd trimester. Antony et al. found that microbiome in placenta was in association with gestational weight gain among women who delivered preterm, but not full-term, with increased abundance of Firmicutes, Actinobacteria, and Cyanobacteria [18]. A recent study found that the relative levels of certain placenta microbiota taxon (genus of Acinetobacter) were lower in gestational diabetes mellitus (GDM), which is a high risk of fetal macrosomia [24]. The data indicates that aberrations in placental microbiota are significantly correlated with the clinical characteristics of the mothers and newborns in macrosomia, which may be due to excess gestational weight gain. However, these results are tantalizing but do not prove causality, further studies are needed.

To our best of knowledge, this is the first study to explore the relationship between placental microbiota profile and fetal macrosomia in neonates. Our study has some notable strengths: 1) Placenta microbiota were successfully identified and isolated. 2) The process of sample collection is rigorously sterile and the microbiota analysis is robust. 3) All the babies were delivered by Cesarean section to minimize potential confounding effects of delivery mode. The study also has some important limitations: 1) Small sample size. Because we excluded GDM to minimize its effects on placental microbiota, it restricted the number of fetal macrosomia recruited. However, only 20 placenta samples were collected from the two groups, which could limit the statistical power and cause potential bias of our study. Next, we aimed at recruiting more participants. 2) Our study only focused on micorbiota composition, but not metagenomic studies. Therefore, we will focus on metagenome in palcenta, and even its correlation with metabonomics. However, our study is novel in providing the evidence of the associations between placental microbiota and fetal macrosomia.

In conclusion, our study showed that a distinct placental microbiota profile is present in fetal macrosomia, and is associated with clinical characteristics of mothers and infants, and umbilical cord blood biochemical parameters. This study contributes to the current knowledge on the putative role of placental microbiota on the increased risks of fetal macrosomia. With the increasing prevalence of fetal macrosomia, a better understanding of the role of placental microbiota can provide critical implications for the development of new strategies for the early prevention of fetal macrosomia, and thus ensure a healthier future for the mothers, infants, and even the young adulthood. Thus, high-quality, large-scale clinical trials and further pre-clinical studies to clarify the underlying mechanisms are urgently warranted.

## MATERIALS AND METHODS

### Ethics and study participants

Informed written consent was obtained from all participants, and the sampling and experimental processes were performed with the approval of the Institutional Review Board and Ethics Committee of Peking Union Medical College Hospital (PUMCH, No: S-002). 20 pregnant women and their full-term newborns (gestational age between 37 and 42 weeks), including 10 fetal macrosomia and 10 newborns with normal birth weight were consecutively recruited and born at Peking Union Medical College Hospital (Beijing, China). Fetal macrosomia is defined as a birth weight of ≥4000 g. It suggested that the wide variation of the sensitivity of ultrasonographic measurement of the fetal weight [25], we accurately weighed all the newborn immediately after birth, which is the most accurate way of diagnosing fetal macrosomia. Several studies have shown distinct effects of delivery mode (vaginal vs. Cesarean section) on the gut microbiota composition of newborn babies [26, 27]. Thus, all the infants delivered by Cesarean section, due to social factors or in response to patient demand, were recruited. The exclusion criteria included: 1) the presence of any antepartum infections and antibiotic treatment during pregnancy; or 2) abnormal glucose metabolism before and during pregnancy, including impaired glucose tolerance, diabetes mellitus and gestational diabetes mellitus; or 3) chronic hypertension, preeclampsia, endocrine disorders and any other severe maternal illnesses; or 4) obstetric risks, such as infants with a gestational age<37 or >42 weeks, multifetation, asphyxia at birth, congenital anomalies, neurological dysfunction, fetal chromosomal anomalies, or inborn errors in metabolism.

### Anthropometric measurements

All the clinical information was collected according to our previous publications [19, 20, 28]. Three trained research nurses and assistants collected clinical characteristics of the mothers and neonates using structured study questionnaires through face-to-face interviews and medical records. Clinical variables of the infants included sex, the mode of delivery, birth weight, body length, ponderal index, head circumference and placenta weight, gestational age, and neonatal complications. Gestational age was defined as the number of completed weeks of gestation, which was determined by the duration of amenorrhoea or was confirmed by an early ultrasound scan during pregnancy. Birth weight and body length of neonates were measured after delivery using a calibrated scale for weight and a measuring board for length. Ponderal index was calculated as birth weight (kg) /body length (m)^3^. Head circumference was measured with a plastic tape to the nearest 0.1 cm. Placenta weight was measured with an electronic scale and determined to the nearest 1 g. Demographic data of the mother included maternal age, body weight and body mass index (BMI) at the beginning of 1^st^ and the end of 3^rd^ trimester, medical history, medications during pregnancy, maternal smoking and diabetes status. The clinical characteristics and anthropometric measurements of each newborn and mother were shown in Supplementary Table 1.

### Umbilical cord blood sampling and analysis

Umbilical cord blood samples were obtained immediately after birth and collected in tubes kept on ice and containing EDTA to prevent clotting. Plasma was obtained by centrifugation (4°C, 3,000 rpm, 10 min) and stored in multiple aliquots at -80°C until analysis. Plasma glucose was analysed by automated glucose oxidase method. Plasma concentrations of insulin were measured by a human insulin-specific radioimmunoassay (RIA) kit (HI-14K, Linco Research Inc., St. Charles, Missouri, USA). The difference between duplicate results of a sample was less than 10% coefficients of variation (CV). Plasma leptin levels were analysed by a human leptin RIA kit (HL-81HK, Linco Research Inc., St. Charles, Missouri, USA). The inter-assay and intra-assay CV were 3.6–6.2% and 3.4–8.3%, respectively. The specificity for human leptin was 100%. Plasma concentrations of insulin-like growth factor-1 (IGF-1) were analysed by a human IGF-1 RIA kit (A15729, Beckman Coulter Diagnostics, Inc., West Sacramento, California, USA). The inter-assay and intra-assay CV were both less than 10% and the specificity for human IGF-1 was 100%. All samples were tested in duplicate and in a blinded manner.

### Placental samples collection

Placental samples were collected by trained pathology personnel, according to the previously described [15, 18]. The placenta samples were collected after childbirth in operating room and the personnel were wearing facial masks and sterile gloves to ensure sterility. Briefly, following standard obstetrical practice, the placenta was delivered and immediately passed off to trained personnel in a sterile container. Then, placenta was weighed and four 1 cm^3^ cuboidal sections were circumferentially excised from separate areas of the placenta, each located 3-4 cm from the insertion site of umbilical cord. To avoid the placenta being contaminated by maternal skin, surrounding atmosphere or any other risks, the placental surfaces of the fetal chorion and maternal decidua were discarded, with only the inner part of the placenta retained. Then, the placental samples were snap frozen in liquid nitrogen and stored for further analysis.

### DNA extraction and 16S ribosomal RNA (rRNA) amplification

Genomic DNA was extracted from placental tissue using the E.Z.N.A.® DNA Kit (Omega Bio-tek, Norcross, GA, USA), following the standard protocol. PCR amplification was performed using 16S rRNA universal primers targeting the V3-V4 region of the bacterial 16S rRNA gene. The primers are 338F 5‘-ACTCCTACGGGAGGCAGCA-3’ and 806R 5‘-GGACTACHVGGGTWTCTAAT-3’, where barcode is an eight-base sequence unique to each sample. PCR reaction mixture were performed in triplicate, consisted of 10 ng template DNA, 2 μL dNTPs (2.5 mmol/L), 0.8 μL forward primer (5 μmol/L), 0.8 μL reverse primer (5 μmol/L), 4 μL 5 × FastPfu Buffer, 0.4 μL FastPfu Polymerase, and ddH2O in a final volume of 20 μL. PCR amplification program was as follows: an initial activation step with 95°C for 3 min, followed by 25 cycles at 95°C for 30 s, 57°C for 30 s, and 72°C for 45 s and a final extension at 72°C for 10 min. Samples that contained no template and those that contained known 16S rRNA gene sequences were used as positive and negative controls in the PCR reactions. Due to the concern for bias in low-abundance samples of placenta tissue, all specimens were extracted and sequenced in triplicate. Sequences from all the three extractions were synthesized for further analysis, as previously described [15].

### 16S rRNA sequencing and microbiota analysis

Amplicons were extracted from 2% agarose gels, purified using the AxyPrep DNA Gel Extraction Kit (Axygen Biosciences, Union City, CA, USA), and quantified using QuantiFluor™-ST (Promega BioSciences LLC, Sunnyvale, CA, USA) according to the standard protocols. Then, purified amplicons were pooled and paired-end sequenced (2 × 300) on an Illumina MiSeq platform (Illumina Inc., San Diego, CA, USA) according to the manufacturer’s instructions. All raw reads were screened according to barcode and primer sequences, using Quantitative Insights Into Microbial Ecology (QIIME, version 1.17), with the following criteria: 1) The 300 bp reads were truncated at any site receiving an average quality score <20 over a 10 bp sliding window; 2) the truncated reads that were <50 bp were abandoned; 3) Sequences that overlap shorter than 10 bp, or reads containing ambiguous characters, or >2 nucleotide mismatch in primer matching were removed. Operational taxonomic units (OTUs) were clustered with the cut-off of 97% similarity using UPARSE (version 7.1, http://drive5.com/uparse/), and UCHIME was utilized to identify and remove chimeric sequences. RDP Classifier (http://rdp.cme.msu.edu/) was used to analyze the phylogenetic affiliation of each 16S rRNA gene sequence, against the silva (SSU115) 16S rRNA database with confidence threshold of 70% [29]. Weighted UniFrac distance metrics analysis was performed using OTUs for each sample, and principal coordinates analysis (PCoA) analysis was conducted according to the matrix of distance [30]. Linear discriminant analysis (LDA) effect size (LEfSe) was used to elucidate the differences of bacterial taxa. The LDA score ≥2 was considered to be important contributors to the model. The cladogram was drawn using the Huttenhower Galaxy web application (The Huttenhower Lab, Boston, MA, USA) via the LEfSe algorithm (http://huttenhower.sph.harvard.edu/lefse/) [31].

### Statistical analysis

Statistical analyses were performed with SPSS 21.0 (SPSS, Inc., Chicago, IL, USA). Results are expressed as mean ± standard deviation (S.D.). Nonparametric variables were mathematically transformed to improve symmetry. Unpaired t-test was used to study differences in continuous variables between groups. The Mann-Whitney U test was used to examine differences in bacterial composition between the two groups. The relation between relative abundance of certain OTU/genus/phylum and clinical information, including anthropometric measurements and umbilical cord blood parameters was performed by Spearman’s correlation analyses. P<0.05 was considered to be statistically significant.

## SUPPLEMENTARY MATERIALS FIGURE AND TABLES



## References

[1] Schwartz R, Teramo KA (1999). What is the significance of macrosomia?. Diabetes Care.

[2] Henriksen T (2008). The macrosomic fetus: a challenge in current obstetrics. Acta Obstet Gynecol Scand.

[3] Stones RW, Paterson CM, Saunders NJ (1993). Risk factors for major obstetric haemorrhage. Eur J Obstet Gynecol Reprod Biol.

[4] Stotland NE, Caughey AB, Breed EM, Escobar GJ (2004). Risk factors and obstetric complications associated with macrosomia. Int J Gynaecol Obstet.

[5] Bergmann RL, Richter R, Bergmann KE, Plagemann A, Brauer M, Dudenhausen JW (2003). Secular trends in neonatal macrosomia in Berlin: influences of potential determinants. Paediatr Perinat Epidemiol.

[6] Morikawa M, Cho K, Yamada T, Yamada T, Sato S, Minakami H (2013). Fetal macrosomia in Japanese women. J Obstet Gynaecol Res.

[7] Bamberg C, Hinkson L, Henrich W (2013). Prenatal detection and consequences of fetal macrosomia. Fetal Diagn Ther.

[8] Hocher B, Slowinski T, Bauer C, Halle H (2001). The advanced fetal programming hypothesis. Nephrol Dial Transplant.

[9] Koyanagi A, Zhang J, Dagvadorj A, Hirayama F, Shibuya K, Souza JP, Gulmezoglu AM (2013). Macrosomia in 23 developing countries: an analysis of a multicountry, facility-based, cross-sectional survey. Lancet.

[10] Dennedy MC, Dunne F (2013). Macrosomia: defining the problem worldwide. Lancet.

[11] Human Microbiome Project Consortium (2012). Structure, function and diversity of the healthy human microbiome. Nature.

[12] Qin J, Li Y, Cai Z, Li S, Zhu J, Zhang F, Liang S, Zhang W, Guan Y, Shen D, Peng Y, Zhang D, Jie Z (2012). A metagenome-wide association study of gut microbiota in type 2 diabetes. Nature.

[13] Watson ED, Cross JC (2005). Development of structures and transport functions in the mouse placenta. Physiology (Bethesda).

[14] Wassenaar TM, Panigrahi P (2014). Is a foetus developing in a sterile environment?. Lett Appl Microbiol.

[15] Aagaard K, Ma J, Antony KM, Ganu R, Petrosino J, Versalovic J (2014). The placenta harbors a unique microbiome. Sci Transl Med.

[16] Pelzer E, Gomez-Arango LF, Barrett HL, Nitert MD (2017). Review: maternal health and the placental microbiome. Placenta.

[17] Doyle RM, Alber DG, Jones HE, Harris K, Fitzgerald F, Peebles D, Klein N (2014). Term and preterm labour are associated with distinct microbial community structures in placental membranes which are independent of mode of delivery. Placenta.

[18] Antony KM, Ma J, Mitchell KB, Racusin DA, Versalovic J, Aagaard K (2015). The preterm placental microbiome varies in association with excess maternal gestational weight gain. Am J Obstet Gynecol.

[19] Zheng J, Xiao X, Zhang Q, Mao L, Yu M, Xu J (2015). The placental microbiome varies in association with low birth weight in full-term neonates. Nutrients.

[20] Xiao X, Zhang ZX, Cohen HJ, Wang H, Li W, Wang T, Xu T, Liu A, Gai MY, Ying S, Schmitz O, Yi Z (2008). Evidence of a relationship between infant birth weight and later diabetes and impaired glucose regulation in a Chinese population. Diabetes Care.

[21] Xiao X, Zhang ZX, Li WH, Feng K, Sun Q, Cohen HJ, Xu T, Wang H, Liu AM, Gong XM, Shen Y, Yi Z (2010). Low birth weight is associated with components of the metabolic syndrome. Metabolism.

[22] Kahyaoglu I, Kinay T, Kayikcioglu F, Kahyaoglu S, Mollamahmutoglu L (2015). Percentage change in body mass index or gestational weight gain: which is a better predictor of foetal macrosomia?. J Obstet Gynaecol.

[23] Li G, Kong L, Li Z, Zhang L, Fan L, Zou L, Chen Y, Ruan Y, Wang X, Zhang W (2014). Prevalence of macrosomia and its risk factors in china: a multicentre survey based on birth data involving 101,723 singleton term infants. Paediatr Perinat Epidemiol.

[24] Bassols J, Serino M, Carreras-Badosa G, Burcelin R, Blasco-Baque V, Lopez-Bermejo A, Fernandez-Real JM (2016). Gestational diabetes is associated with changes in placental microbiota and microbiome. Pediatr Res.

[25] Walsh JM, McAuliffe FM (2012). Prediction and prevention of the macrosomic fetus. Eur J Obstet Gynecol Reprod Biol.

[26] Dominguez-Bello MG, Costello EK, Contreras M, Magris M, Hidalgo G, Fierer N, Knight R (2010). Delivery mode shapes the acquisition and structure of the initial microbiota across multiple body habitats in newborns. Proc Natl Acad Sci U S A.

[27] Mackie RI, Sghir A, Gaskins HR (1999). Developmental microbial ecology of the neonatal gastrointestinal tract. Am J Clin Nutr.

[28] Zheng J, Xiao X, Zhang Q, Mao L, Li M, Yu M, Xu J, Wang Y (2014). Correlation of high-molecular-weight adiponectin and leptin concentrations with anthropometric parameters and insulin sensitivity in newborns. Int J Endocrinol.

[29] Amato KR, Yeoman CJ, Kent A, Righini N, Carbonero F, Estrada A, Gaskins HR, Stumpf RM, Yildirim S, Torralba M, Gillis M, Wilson BA, Nelson KE (2013). Habitat degradation impacts black howler monkey (Alouatta pigra) gastrointestinal microbiomes. ISME J.

[30] Lozupone C, Hamady M, Knight R (2006). UniFrac--an online tool for comparing microbial community diversity in a phylogenetic context. BMC Bioinformatics.

[31] Segata N, Izard J, Waldron L, Gevers D, Miropolsky L, Garrett WS, Huttenhower C (2011). Metagenomic biomarker discovery and explanation. Genome Biol.

