# Supplementary material for: Correlation of placental microbiota with fetal macrosomia and clinical characteristics in mothers and newborns
Source: Oncotarget. 2017 Jul 18;8(47):82314–25. doi: 10.18632/oncotarget.19319 (PMC5669892; doi:10.18632/oncotarget.19319)
Supplement: Supplementary file 1 [file oncotarget-08-82314-s001.pdf]

Correlation of placental microbiota with fetal macrosomia and clinical characteristics in mothers and newborns

SUPPLEMENTARY MATERIALS

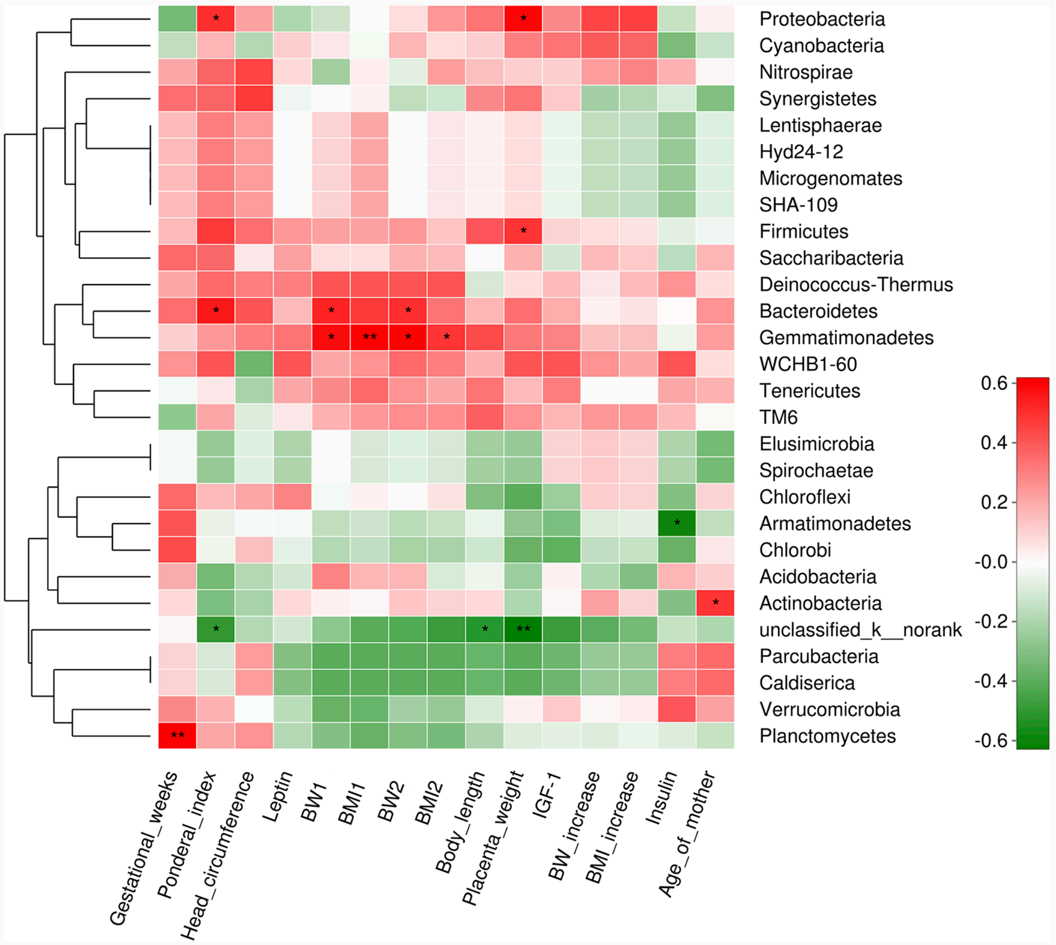

Supplementary Figure 1: Correlation analyses between identified phyla of placental microbiota and clinical parameters. Correlation analyses between relative abundance (%) of specific phylum and clinical parameters were performed by using Spearman's correlation analyses. n = 10, in each group. (*) represents the specific phylum whose abundance were significantly correlated with certain clinical parameters. The color of the spots in the right panel represents R-value of Spearman's correlation between the phylum and clinical parameters. BW, body weight; BMI, body mass index; BW1, body weight at the beginning of 1st trimester; BMI1, BMI at the beginning of 1st trimester; BW2, body weight at the end of 3rd trimester; BMI2, body mass index at the end of 3rd trimester; IGF-1, insulin-like growth factor-1.

Supplementary Table 1: Clinical characteristics and anthropometric measurements of each newborn and mother

Infants										Mothers						Umbilical cord blood		
**IDs**	**Sex**	**Mode of delivery**	**Birth weight (g)**	**BL (cm)**	**PI (kg/m3)**	**HC (cm)**	**PW (g)**	**GA (wks)**	**Age (yrs)**	**BW1**	**BMI1**	**BW2**	**BMI2**	**BW↑**	**BMI↑**	**Insulin (µU/mL)**	**Leptin (ng/mL)**	**IGF-1 (ng/mL)**
**Ctl 1**	F	C-section	3030	48	27.4	35	540	39.29	38	45	17.58	59.5	23.24	14.5	5.66	20.74	4.96	31.1
**Ctl 2**	F	C-section	3170	48	28.66	32.8	620	38.57	28	50	19.53	65	25.39	15	5.86	7.29	14.53	60.42
**Ctl 3**	M	C-section	3120	50	24.96	34.5	545	39	42	62	21.97	78	27.64	16	5.67	10.41	10.9	40.39
**Ctl 4**	F	C-section	3200	51	24.12	32	550	41	26	49	19.14	62	24.22	13	5.08	13.62	12.57	35
**Ctl 5**	M	C-section	3010	49	25.58	34	600	39	27	56	20.08	75	26.89	19	6.81	9.71	7.35	67.48
**Ctl 6**	F	C-section	3110	49	26.43	33	650	38.86	32	47	17.91	70	26.67	23	8.76	10.32	4.03	64.09
**Ctl 7**	M	C-section	3180	48	28.75	34.9	630	39	33	51	19.2	65	24.46	14	5.26	7	3.88	25.5
**Ctl 8**	F	C-section	3200	50	25.6	34	725	38.43	29	69	23.32	80.5	27.21	11.5	3.89	17.68	5.69	38.98
**Ctl 9**	M	C-section	3200	50	25.6	34.5	640	38.43	32	56	19.84	76	26.93	20	7.09	21.32	6.5	57.92
**Ctl 10**	M	C-section	3250	49	27.62	34	520	38.72	35	50	19.53	64	25.00	14	5.47	13.31	4.56	98.97
**M1**	F	C-section	4775	52	33.96	32.5	1150	40.43	30	65	23.31	88.5	31.73	23.5	8.42	50.18	26.9	237.21
**M2**	M	C-section	4680	53	31.44	37.5	1065	40.57	27	52.5	20	67.5	25.72	15	5.72	17.37	9.16	91.86
**M3**	M	C-section	4640	55	25.23	34	985	38	36	67	22.65	86	29.07	19	6.42	17.31	9.12	114.7
**M4**	M	C-section	4510	52	32.07	34.8	955	38	28	54	20.32	80	30.11	26	9.79	14.68	8.78	41.63
**M5**	M	C-section	4410	51	33.25	35	850	39.72	29	61	23.24	76	28.96	15	5.72	9.28	9.96	52.5
**M6**	F	C-section	4370	53	29.35	33.6	940	39	32	67	24.31	83.5	30.30	16.5	5.99	18.98	14.15	100.13
**M7**	F	C-section	4290	52	30.51	36	760	40.86	29	58.5	22.02	82.5	31.05	24	9.03	7.12	24.06	36.78
**M8**	M	C-section	4240	50	33.92	35.5	850	40.29	30	74	30.02	91.5	37.12	17.5	7.10	19.8	21.04	77.52
**M9**	M	C-section	4220	52	30.01	34	985	38	29	48	20.5	75	32.04	27	11.54	26.63	12.37	96.93
**M10**	M	C-section	4280	52	30.44	34.2	870	38	36	60	21.26	85	30.12	25	8.86	25.31	12.88	82.7

Ctl, control; M, macrosomia; F, female; M, male; C-section: Cesarean section; BL, body length; PI, Ponderal index; HC, head circumference; PW, placenta weight; GA, gestational age; Wks, weeks; Yrs, years old; BW, body weight; BW1, body weight at the beginning of 1st trimester; BW2, body weight at the end of 3rd trimester; BMI, body mass index; BMI1, BMI at the beginning of 1st trimester; BMI2, body mass index at the end of 3rd trimester; BW↑, BW increase during pregnancy; BWI↑, BWI increase during pregnancy; IGF-1, insulin-like growth factor-1.

Supplementary Table 2: Phylotypes significantly different at the genus level

	Control (proportions)	Macrosomia (proportions)	P value
**Novosphingobium**	0.000%	0.079%	0.008**
**Achromobacter**	0.048%	0.168%	0.021*
**Geothrix**	0.026%	0.000%	0.024*
**Acinetobacter**	0.142%	1.564%	0.024*
**Paracocccus**	0.000%	0.328%	0.045*
**Pseudonocardia**	0.000%	0.905%	0.045*
**Woodsholea**	0.000%	0.347%	0.045*
**Nitratireductor**	0.006%	0.437%	0.048*
**Pelomonas**	0.002%	0.166%	0.049*
**Prevotellaceae_NK3B31_ group**	0.448%	0.113%	0.028*
**Dechloromonas**	0.065%	0.008%	0.085
**Lactococcus**	0.192%	0.644%	0.052
**Faecalibacterium**	0.023%	0.728%	0.081
**Sphingomonas**	0.178%	0.318%	0.088
**Bacteroidales_S24-7_ group_norank**	4.584%	2.621%	0.092
**Gemmatimonadaceae_ uncultured**	0.001%	0.609%	0.055
**Bacteria_unclassified**	53.290%	21.341%	0.003**
**Alcaligenaceae_unclassified**	0.949%	3.311%	0.005**
**Gallionellaceae_ unclassified**	0.006%	0.000%	0.082
**Lachnospiraceae_ unclassified**	0.119%	0.717%	0.084

Data of placental microbiota were showed as relative abundance (%) of genera in each group. Statistical analysis was performed by the Mann-Whitney U test. The comparisons of genera with no statistically significant difference ($P \geq 0.1$) were not shown. n = 10, in each group. *$P < 0.05$, **$P < 0.001$ macrosomia vs. control group.
